# Striving for a balance between leading and following the patient and family – nurses’ strategies to facilitate the transition from life-prolonging care to palliative care: an interview study

**DOI:** 10.1186/s12904-018-0311-7

**Published:** 2018-04-03

**Authors:** Ulrika Hilding, Renée Allvin, Karin Blomberg

**Affiliations:** 1 Örebro University Hospital, Örebro, Sweden; 2 Clinical Skills Centre, Örebro University Hospital, Örebro, Sweden; 3 Faculty of Medicine and Health, School of Health Sciences, Örebro University, S-70182 Örebro, Sweden

**Keywords:** Interviews, Palliative nursing care, Qualitative research

## Abstract

**Background:**

The transition from life-prolonging to palliative care (PC) can be challenging often characterized by psychical, physiological, social and existential changes. Knowledge of how to support the patient and family in this specific care phase is lacking, and this area needs to be further explored. The aim of this study was to investigate strategies that registered nurses (RNs) use to ease the transition from life-prolonging care to PC for patients with incurable disease.

**Methods:**

The study has a descriptive design. Fourteen RNs working in a specialized PC unit were interviewed. The data were analysed using content analysis.

**Results:**

The RNs’ strategies can be described under the categories “Getting to know the patient and creating a relationship”, “Providing support”, “Adapting to individuals’ needs” and “Enabling conversations”.

**Conclusion:**

The findings show that the RNs in this population used strategies that not only took time but also required knowledge about the transition process and required the ability to identify and meet patients’ and families’ unique needs. Patients’ difficult and exposed situation needs to be addressed through a structured follow-up after informing about the change from life-prolonging care to PC. RNs have a unique role of supporting both the patient and the family in the transition from life-prolonging care to PC for patients with incurable disease.

## Introduction

Palliative care (PC) has changed during the last decades and today the patient and family are involved in the PC process in a much more direct way compared with previously. The patient’s role has been strengthened, as the health care team are obliged to inform patients of their prognosis, be open for a discussion about any change in goals and plan the care in line with the individual patient’s wishes [1]. The end of a person’s life is often characterized by physiological, social and existential changes due to loss of roles and functions that effect self-image [2–4], often occurring in parallel with physical suffering [5]. This situation is challenging, and the patient and their family need to be supported. However, knowledge of how to support the patient and family in this specific phase is lacking, and this needs to be further explored.

## Background

The transition to PC is associated with the experience of leaving a predictable and entering a new, unpredictable situation [6]. Family caregivers describe the transition to PC as bringing a constant awareness of death, which increases in times of deterioration of symptoms or when waiting for test results or information from the doctors [7]. Studies show that information about incurable cancer and impending death can triggered existential issues and angst [2] and the transition from discussing a cure to enter PC can cause emotional distress to the patient, the family and even health professionals [8, 9]. At this point the patient and family enter a very special and vulnerable time and the role of registered nurses (RNs) is to support them during this process.

Several studies have described the importance of receiving honest and sincere information about life being limited. This communication enables the patient and their family to prepare and plan for the end of life and focus on what is important in the remaining time. This is a meaningful and valuable part of the grieving process of the family, both during the PC and after the death [1]. Kirby et al. report that Australian palliative specialists find families’ situation critical when it comes to transitioning to PC [10]. They stress the importance of a new focus on training and education, including cultural competence education, of health professionals to enable a more comprehensive understanding of the patient’s family and the challenging changes in their needs.

In Sweden, PC is based on WHO’s definition [11], as a means to improve the quality of life of patients facing life-threatening illness and of their families, regardless of diagnosis or age, including both at an early stage of serious illness and the late phase of end-of life. PC is not merely given in a specialized palliative clinic, but also in general care such as municipal care or at hospitals, where RNs have a central role. The Swedish guidelines [12] emphasize the need to inform the patient and family when the medical situation changes and cure no longer is an option. The information may be summarized by the treating doctor. Crucial for the upcoming care is that the patient and family understand the new direction of care.

Warnock emphasizes that transitions in health care constitute time points and a process where the RN has an important role, of taking part in giving information, and preparing and helping the patient to receive and cope with the information [13]. In describing the role of the RN and the challenges in breaking the bad news Griffiths et al. show that, of all the health care workers, RNs are the ones who are close to the patient and their family during the final period of life [14]. They have to face with patient’s existential issues and angst. However, they often feel unprepared and are in need of tailored education to be able to give optimal care at the end of life [14].

To summarize, the complex transition to PC is not fully explored, and the transition to PC distinguishes from other care transitions as it includes; the emotional component in facing death, the concern of timing the transition, and patient’s and family’s understanding of the process [9]. There is a lack of studies that focus on the role of the RN and the strategies RNs use to facilitate the transition to PC. Increased knowledge would give a better understanding that could contribute to development of supportive care in this phase of the illness trajectory irrespectively the disease has evolved over years or is a sudden change in health. Therefore, the aim of this study was to describe strategies RNs employ in facilitating the transition from life-prolonging care to PC for patients with incurable disease.

## Method

The study has a descriptive design [15]. Individual interviews were conducted with RNs working in a hospital-based specialized PC unit, including both inpatient care and advanced home care, at a university hospital in Sweden. Ethical approval was obtained from the Regional Ethics Board in Uppsala (Reg.no. 2013/244).

### Participants and procedure

A purposeful sampling was used to include RNs with a range of experience of the research question [15]. The inclusion criterion was RNs with at least 1 years’ experience of working in PC. Fifteen RNs, selected by the manager of the unit, were personally asked to participate in the study. All participants gave written informed consent. A total of 14 RNs were interviewed, one man and 13 women 27–64 years old (median 43 years). They had between 2 and 32 years’ experience of working in PC. One worked only night hours, four worked both night and day shifts, and nine only worked during daytime.

### Data collection

Individual semi-structured interviews [16] were conducted by the first author using an interview guide designed according to the aim of the study. The interviews started with an open-ended question: “How do you facilitate the transition from life-prolonging care to palliative care for your patients?” Afterwards more specific questions were asked, for example: “Can you explain how you follow up after breaking the bad news to the patient and their family?” To reach a deeper understanding, probing questions were asked, such as “Could you describe that in more detail?”

Two pilot interviews were conducted to test the interview guide. This resulted in a few revisions of the questions by changing the initial question to give the RNs even more opportunity to speak freely. These interviews were included in the data analysis. Demographic data were collected at the beginning of each interview. All RNs chose to be interviewed during their scheduled time at their workplace. The interviews were audio-recorded and lasted between 20 and 60 min. All the interviews were transcribed verbatim.

### Data analysis

The data were analysed using qualitative content analysis [17]. As a first step, the transcribed interviews were read thoroughly several times to get an overview of the content, and to identify meaning units supporting the aim of the study. The meaning units were condensed to give a more manageable material without losing the essence of the text. Out of the condensed units, codes were extracted, consisting of words or a short sentence. For example, codes might represent specific actions or an approach that the RNs described in the interviews. During the analysis, we attempted to create codes that were close to the text, although more concise as well as abstracted to a higher logical level (see Table 1). To minimize the risk of interpretation errors the researchers paid special attention to the context of the meaning unit during the condensing and coding process. The codes were sorted into subcategories based on similarities and differences in the RNs’ strategies. From the subcategories, four categories were abstracted describing RNs’ strategies to facilitate and ease the transition process for patients. Finally, the underlying meaning (i.e. the latent content) was formulated into a theme. During the analysis, discussions about categorization were held between all researchers to increase trustworthiness. In the following, quotations have been used to illustrate the findings.

**Table 1:** Example of a meaning unit, condensed meaning unit and codes

Meaning unit	Condensed meaning unit	Codes
… still, I strive to be as honest as possible all the time, you strive towards being honest and telling what you maybe think they need	… strive to be as honest as possible. Strive towards telling what you think they need.	*Nurse strives to be as honest as possible.* *Nurse tells what she thinks patients need.*

## Results

The analysis resulted in the theme “*Striving for a balance between leading and following the patient and family*”. The theme is based on four main categories (presented below) depicting the RNs’ descriptions of strategies used to facilitate the transition from life-prolonging care to PC (Table 2).

**Table 2:** The final subcategories, categories and theme

Subcategories	Categories	Theme
Getting close to the patient	Getting to know the patient and creating a relationship	Striving for a balance between leading and following the patient and family
Exploring where the patient is emotionally		
Involving family		
Identifying and meeting needs	Providing support	
Informing and explaining		
Emotional support		
Family support		
Responsiveness	Adapting to individuals’ needs	
Adherence		
Consideration and respect		
Facilitating talk	Enabling conversations	
Reality checking		

The theme can be understood as the core of the RN’s work during the specific phase of a patient’s life. During this period, the RN cautiously tries to reach out to the patient and their family, and to find out how well informed they are about the patient’s situation. The RNs also tries to establish how the information about the illness and its progress is comprehended. The RNs described how they took repeated initiatives to invite the patient and their family to dialogue. During the progress of the illness the RNs were available to meet and satisfy patients’ differing needs. They strived to show respect and share concerns, at the same time as they gently tried to build reality awareness.

*… do you see … how ill your mother is? That it’s different from yesterday? So maybe tell the daughter that her mother can’t keep the food; she is that ill … it’s not possible to be much more ill than this. (RN 10, inpatient care)*

### Getting to know the patient and creating a relationship

To build trust and facilitate dialogue, the RNs strived to get to know the patient and the family. The RNs considered physical, psychological, social, and existential aspects during this process. They focused on seeing the person behind the illness and what patients considered as most important. When the time of care was short, or when the RNs did not meet the patient before the illness had advanced, it was described more difficult to establish a relationship with the patient and family.

One of the RNs described how she wanted to be confident with the patient before speaking about bad news. For her, the point in time for initiating existential matters was crucial. Mutual interests were used to start a dialogue and create a common base. One RN described how she actively directed the dialogue to progressively turn to what was important. If the patient was giving vague answers this was a complicating factor. According to the RNs, this was hard, because it might lead to misunderstandings. The RN was then unsure whether the patient understood more than they showed – or whether the opposite applied. To find out what, and how much, a patient wanted to know was seen as a delicate task.

*… even if you have this diagnosis you might not want to know all about it, and how medically literal can you be? … it might sound pretty hard if you read from a medical record. You have to choose your words. (RN 7, advanced home care)*

Some of the RNs described themselves as straightforward when getting to know the patient. Others tried to be more cautious and careful.

… *sort of try to listen and see where the person is … (RN 11, inpatient care)*

The RNs generally considered that family participation eased the patient’s situation. The RNs took care of giving the family the opportunity to prepare for the loss of a family member. If the patient agreed, the family were given the opportunity to receive information, and to take part in making decisions. However, RNs described that sometimes the family expressed a denial to the patient’s situation and were unable to take in information. These situations were described as a challenge for the RNs in order to establish a relationship to the family.

### Providing support

Different kinds of support both to the patient and the family was described as central. The support was given in different ways, depending on the patient’s symptoms and situation. One type of support involved identifying and meeting patient needs of symptom relief, and coordinating different actions to deal with both medical and social issues. The RNs also provided information about the care of the patient, the course of the illness, and the plans for patient and family. The type of information they handled varied, but mostly concerned answering questions from patients and family. They explained what was going on with the patient. Sometimes they had to give information without being asked any questions. The RNs described how they strived to be responsive, and give information in manageable amounts, being well aware that some patients did not want to be fully informed. Therefore, to give support and mediate a sense of safety, all patients had to be asked about how much they wanted to know about their illness and its progress. To be able to follow up after bad news, exploring previously given information and how it was perceived, was described as central. The RNs used different strategies to find out how well informed the patient was, and how well they understood the situation. Several RNs noted that sometimes the patient and/or family did not grasp the information although it was given repeatedly. One of the RNs described that just *knowing* that bad news had been received made it easier for her to communicate with the patient. She valued having something to relate to in her conversations with the patient and family.

*… you must help the patient in all ways, you should relieve symptoms* – *focus on that instead of curative treatment. (RN 8, inpatient care)*

Support was provided through listening to the patient and letting them talk, as well as confirming and meeting different emotions. To give time and be there for the patient was described as emotional support. One of the RNs described how she let her patients pass through their stages of anger and guilt just by following them in the process. Others described taking one day at a time with their patients. Hope was considered to be precious to the patients, and the RNs strived to let them preserve their hope.

*I don’t want to take all hope away, because this hope might be the one and only reason why they wake up one more morning. (RN 13, advanced home care)*

The RNs tried to help the patients to focus on what could be done, instead of what could not. It was considered easier for both the patient and the family if the patient accepted the situation and the RNs described that these patients often experienced a better last period.

Support was also given to the family, which the RNs saw as equally important to giving support to the patient. This support covered both practical information and supportive conversations. One RN stressed the importance of relieving the family from guilt with respect to the patient. She reported that many family members expressed conflicting interests, and did not feel that they were as physically present as they would have wanted. The RNs tried to ease their burden by reassuring them that they were looking after the patient, and that the family members could come and go as they wanted and were able.

*… you have to release them from debt, help them to not feel guilty. (RN 12, inpatient care)*

The RNs understood that family members who took care of the patient in their home mostly needed practical support from the home care service and/or the occupational therapist; but they also needed confirmation that they were going through a hard time.

### Adapting to individuals’ needs

The RNs strived to be responsive by showing respect and adapting to individual patients’ needs. A strategy they used was to be clear about the meaning of the concept PC. The RNs on the ward made great efforts to *see* the patient as an individual, and also the family, and to make them feel as if they were at home. Being available and giving enough time to the patient and family were seen as important. Considering the follow up after the bad news, all RNs described that having an empathetic approach was of utmost importance, although the initiatives they took varied depending on individual patients and families. The RNs also showed patients and their families consideration and respect when existential questions were discussed. They described how they tried to meet individual needs:

*… sort of invited to help during the hardest times and then you are open-minded and try to meet people and accept them the way they are. (RN 12, inpatient care)*

Flexibility was considered important and the RNs adapted their actions based on factors such as patients’ diagnosis, personality and age. Nurses used different tactics to make a hard situation easier, sometimes tried distraction without detracting from the seriousness of the situation. One RN told that she tried to use humor when appropriate:

*Even if you are dying, you have to laugh and often you can actually attract it, even in the most difficult, sad situations... And it’s liberating … you may think things are fun yet. You cannot just sit and think about dying. You live today, today we are here, and do not be afraid to laugh…That (humor) is an weapon, even for our patients (RN 3, advanced home care)*

### Enable conversations

The RNs took a proactive approach when talking to their patients. Asking open-ended questions and posing follow-up questions when patients initiated talk were common strategies. They facilitated conversations through catching the right moment. One RN used to initiate conversations while she performed her everyday practice, without focusing on the talk itself. Patients’ understanding of bad news information was eased through conversations, so it was important not to back off from difficult talks. Sometimes the RNs would bounce questions back to the patients, by asking about their feelings and thoughts. Also, it was important to make it easier for patients and families to start talking. One RN made a point of paying extra attention to patients with an introvert personality:

*You have to try to catch people* – *invite yourself. They can be shy or something. (RN 12, inpatient care)*

Several of the RNs described how patients and family often tried to protect each other, and that it could be difficult for them to speak openly in front of each other. The RNs emphasized the importance of making the patients understand that they could be totally honest with them.

*… because he doesn’t have to pretend to us, but maybe he has to pretend in front of his wife and daughter. (RN 3, inpatient care)*

Conversation was also used to give the patient and family a reality check about the illness and its progress. According to the RNs, this was essential in families where neither part took the situation in. The RNs tried to be empathetic, albeit honest in their conversations, and repeated the information several times because these patients and/or their families could have difficulties to take in or understand all the information at the same time. Some RNs stressed the importance of being totally honest to create a trust between the patient and the RN. It was described as important not to pretend or making false promises to the patient. A balance between respecting patients’ defence mechanisms and the RNs need to be honest was described.

*They don't want anything else and then I think that one has to step back. But on the other hand, you can't promise anything: never make promises. (RN 3, inpatient care)*

## Discussion

The findings show the complexities and dynamics of RNs’ work to facilitate patients’ transitioning between life-prolonging care and PC. The RNs in this study said they aimed for a balance between leading and following the patient and family. Their striving towards this balance can be seen as central to RNs’ work with patients who have just received information regarding their disease and the change in care from life-prolonging care to PC. The strategies the RNs used to facilitate transition were getting to know and creating a relationship with the patient, providing different forms of support, for example through enabling conversations, and taking different approaches to individuals’ needs, which could seen as central for providing PC. In a study about experiences of comfort among persons in need of PC, Coelho et al. found that patients felt more comfortable when healthcare was based on compassion, hope, interpersonal, transpersonal and intrapersonal relationships [18].

The findings further show that RNs used a range of strategies, but all RNs stressed that they always focused on what was best for the patient to facilitate the transition between life-prolonging care and PC. It has been noted that RNs are less able to meet a patient’s needs if the patient is not participating, or where communication is insufficient [19, 20]. In the present study the importance of responsiveness and respect for the patient’s wishes concerning prognostic information was emphasized. The patient’s needs for information can vary, ranging from a wish to know nothing at all to wanting to know all the details. According to Reinke et al. RNs give prognostic information by establishing what the patient already knows and then based on the patient’s wishes regarding the information [19]. The findings from this study showed same strategies, but some ethical dilemmas were mentioned, such as when the patient had one wish and the family another. This was described as a challenge and difficult to handle by the RNs. This need to be further explored for understanding of how RNs dealt with these dilemmas to further improve education and practice in PC.

Benzein et al. describes the characteristics and means of hope of persons with advanced cancer [21, 22]. Hope can be described as reconciliation with life and death and is central in palliative care [21–24]. Reconciliation was something the RNs in the present study described as an important task to help patients with. One strategy RNs used was to find out where the patient was emotionally and how the information they had been given had been received. This was done during conversations focus on the impending death, where the patient was invited to take the lead, both regarding the content and in terms of time. This finding highlights the need for creating a space, a culture where the patients and their families feel safe to express existential concerns as well as aspects of spirituality [2, 6, 25]. As mentioned, the RNs tried to meet every individual’s wishes with respect to what information and how many details patients wanted. According to Griffiths et al. it is not only hard to determine what information to give but it is also difficult to judge when is the right time for information [14]. To give prognostic information too early could “close doors” to further talk – and to give it too late could mean that there is no time to prepare for death. Even if the essential of communication in PC is highlighted in literature and guidelines, errors related to PC are experienced by patients as communication and information errors and errors associate to trust/empathy. Warnock has put down guidelines and advice for giving patients’ bad news [13]. One of the guidelines stresses, as the RNs in the present study have done, that it is important to have both formal and informal talks. It is not always pre-booked and planned time that provides conditions for the best talk. Instead, staff have to make the most of the times when the patient or family hint they want to talk.

To explain different events to the patient and the family, as well as facilitate families’ participation, both in the talks and in the care, were other examples of ways to give support. Bloomer et al. confirm that family participation in planning end of life care is sufficient to give the patient the care he or she wants [26]. A family that is involved can also give the sense of meaning and closeness to the patient. RNs thought that planning end of life care is a process taking place during several meetings where patient and family attendance is a necessity. In our findings, RNs perceived it to be important to involve the family and give them support. Discussion of prognostic uncertainty is of importance for family members [27]. Often, the RNs wished for more open communication between the patient and the family, but they met barriers. They described guiding the patient and family through the challenging and vulnerable phase by being responsive, considerate and respectful; and by being honest while giving continuous support. Lundberg et al. state that a family’s emotional experience of support is more important than the support itself [28].

Registered nurses are uniquely positioned to support patients during this difficult time of transition. It encompasses not only what the RNs *do*, but also how they *see* the person in need of palliative care [29]. However, support given by health care personnel is valuable only when considered so, which suggests that RNs may need to adapt their strategies further and, moreover, that they need to strive to evaluate their strategies in consultation with the individuals in their care and their families.

### Strengths and limitations

To increase trustworthiness of the study we strived for detailed descriptions of the study context, selection, participants’ characteristics, data collection and analysis. The findings are illustrated with appropriate quotes to further enhance trustworthiness [15, 17]. Data collection was done in a specialized PC unit. This needs to be taken into consideration when translating the findings to contexts outside of specialized care units, but our findings are probably applicable even to general PC. If not transferable, the findings could hopefully even give some support and guidance when caring for patients with incurable diseases outside PC. However, we used a qualitative design with the intent to seek information on and understanding of RNs’ strategies, rather than make generalizations to a larger population. In the analysis, continuous discussions about how to categorize the findings were held between all the authors. The authors have different expertise (including clinical expertise in PC, intensive care and anaesthesiology, as well as education and research), which gives different perspectives and opportunities for analytical discussions of the findings.

## Conclusion

Patients’ difficult and exposed situation needs to be addressed through a structured follow-up after informing about the change from life-prolonging care to PC. Registered nurses have a unique role of supporting both the patient and the family in the transition from life-prolonging care to PC for patients with incurable disease. RNs use strategies that require understanding and knowledge of the transition process, the ability to recognize and understand patients’ unique needs concerning the transition, and experience of how to meet those needs. Furthermore, RNs need to adapt their strategies and to strive to evaluate them in consultation with the individuals in their care and their families.

### Clinical implications

Patients’ difficult and exposed situation during the transition from life-prolonging care to PC needs to be addressed. Studies have shown that RNs often meet patients or families who have not fully understood the information that they have been given or why the decision to transition to PC has been made. Guidelines to help increase patients’ understanding and participation in decision making would improve the situation of patients and families. The findings of this study could be used in educational settings and discussions with students, newly employed nurses and RNs working outside of specialized units. In this way, optimal care for PC patients and their families may be achieved.

## Abbreviations

PC: Palliative Care
RN: Registered Nurse
RNs: Registered Nurses
